# Electromigration Analysis for Interconnects Using Improved Graph Convolutional Network with Edge Feature Aggregation

**DOI:** 10.3390/mi15081046

**Published:** 2024-08-18

**Authors:** Ruqing Ye, Xiaoming Chen

**Affiliations:** 1 School of Integrated Circuits, Dalian University of Technology, Dalian 116024, China; mie32@mail.dlut.edu.cn; 2 School of Optoelectronic Engineering and Instrumentation Science, Dalian University of Technology, Dalian 116024, China

**Keywords:** electromigration, hydrostatic stress, graph convolutional network, interconnect

## Abstract

Electromigration (EM) is a critical reliability issue in integrated circuits and is becoming increasingly significant as fabrication technology nodes continue to advance. The analysis of the hydrostatic stress, which is paramount in electromigration studies, typically involves solving complex physical equations (partial differential equations, or PDEs in this case), which is time consuming, inefficient and not practical for full-chip EM analysis. In this paper, a novel approach is proposed, conceptualizing circuit interconnect trees as a graph within a graph neural network framework. Using finite element solution software, ground truth hydrostatic stress values were obtained to construct a dataset of interconnected trees with hydrostatic stress values for each node. An improved Graph Convolutional Network (GCN) augmented with edge feature aggregation and attention mechanism was then trained employing the dataset, yielding a model capable of predicting hydrostatic stress values for nodes in an interconnect tree. The results show that our model demonstrated a 15% improvement in the Root Mean Square Error (RMSE) compared to the original GCN model and improved the solution speed greatly compared to traditional finite element software.

## 1. Introduction

Since the debut of the first integrated circuit, the field has seen rapid advancements. The journey from Small-Scale Integration (SSI) to Very-Large-Scale Integration (VLSI) tracks a near-exponential growth in transistor counts and equally swift progress in semiconductor technology, now achieving 5 nm processes and beyond. While performance has surged, new challenges have emerged [1,2,3,4]. Despite a higher circuit density and performance now being attainable, vulnerability to various aging mechanisms that affect everything from front-end to back-end processes is also becoming a factor, seriously affecting the reliability of the circuit. Reliability is very important for the chip, determining whether the chip can operate properly according to the design expectations, and may also affect the life of the chip, leading to early loss of performance or even complete failure.

Electromigration (EM) is one of the major reliability issues for interconnects in a circuit, and the significance keeps growing as technology nodes continue to advance. EM affects integrated circuits primarily by impacting their interconnects, which can be categorized into two types based on their functions. Signal interconnects serve to connect different regions of the chip, facilitating accurate signal transmission to various registers. Power Grid (PG) network interconnects are crucial for delivering voltage from the power pads, thereby powering the chip. Both types of interconnects must operate correctly for the chip to function reliably and steadily. Notably, the PG network is more vulnerable to EM than signal lines due to its exposure to unidirectional currents. In contrast, signal lines carry bidirectional or pulsed currents. Additionally, the current density in PG networks is typically higher than that in signal lines. Under the influence of EM, the sustained high current flow in one direction continuously causes stress accumulation at the ends of the interconnects, meaning that EM causes the PG network to fail more easily [5].

Research focusing on EM analysis has been performed by various scholars. Early studies on EM primarily utilized the Black model [6,7], which estimates the Mean Time to Failure (MTTF) for interconnects, facilitating EM analysis for simple design structures. However, research has indicated that this model tends to be overly conservative and is generally only applicable to basic interconnects [8,9]. This limitation is particularly significant in the analysis of PG networks, where the conservative estimates provided by the Black model are noticeably excessive.

Given these limitations, recent research has shifted focus towards the Korhonen model [10], which incorporates a physically based analysis of EM. For instance, Huang et al. [11] employed the Korhonen model, accounting for factors such as the current density, temperature, and stress gradients, to develop a more accurate and physically relevant EM analysis model. Following this approach, Chatterjee et al. [12] used this model to predict the lifetime of PG networks, finding that their lifespans are approximately 2.75 times longer than those predicted by the Black model. These findings highlight the conservative bias of the Black model and demonstrate the greater accuracy and applicability of the Korhonen model for more complex situations. Solving the Korhonen equation is essential for conducting physically based EM analysis, which requires dealing with partial differential equations (PDEs). Efforts to find numerical and analytical solutions have been moderately successful but are significantly demanding in terms of computation and time, leading to a serious limitation in practical use [5,11,12,13,14,15,16,17].

In order to solve the problem caused by computational difficulties, various solutions have been proposed. Najm et al. [18] proposed to convert the circuit into an equivalent RC circuit, given that the dynamic behavior of the stresses and fluxes in metal lines is exactly identical to the dynamic behavior of voltages and currents in certain RC circuits, making it possible to carry out EM analysis indirectly for metal interconnects. Chen et al. [19] proposed a new semi-analytical transient stress analysis method to concurrently consider the effects of EM and thermomigration (TM) on multi-segment interconnects, solving the coupled EM-TM partial differential equations using the separation of variables method to obtain analytic solutions for steady-state temperature and stress distributions. However, the methods above still have problems of insufficient efficiency and application range.

As machine learning continues to evolve, its potential in the field of EM analysis is becoming increasingly evident. Sukanta et al. [20] introduced a machine learning approach for the first time to predict EM-aware aging in PG networks, which significantly speeds up EM assessment compared to traditional methods and achieves superior or comparable MTTF predictions against existing state-of-the-art models. The analytical basis used for this method is the Black model, which has limitations in its accuracy and scope of application. Jin et al. [21] developed a fast transient hydrostatic stress analysis method using generative adversarial networks (GANs) for EM failure assessment in multi-segment interconnects, inspired by the image synthesis features of deep neural networks, which can produce accurate stress distributions rapidly for EM analysis. This method took effect on fixed-size graphs. Zuo et al. [22] proposed a model based on optimized backpropagation neural networks, which can predict the transient EM stress of three typical wires of different sizes and at different current densities. The only three interconnect structures for which this method is applicable are straight three-terminal wires, t-shaped four-terminal wires and cross-shaped five-terminal wires, which have a limited range of applicability. Saleh et al. [23] proposed a neural network with a look-up table for void dynamics simulations in electromigration analysis. Hou et al. [24] introduced a feed-forward neural network (FNN) to solve the stress-based partial differential equations. Due to the limitation of computational cost, the method mainly worked on interconnects with simpler structures. Lamichhane et al. [25] proposed an approach based on physics-informed neural networks (PINNs), which achieved speedup compared to an FEM solver. The method had relatively accurate results, although it required a considerable amount of computational time, particularly in cases with a higher number of branches.

To address the issues of lengthy computation time, limited applicability and high computational cost associated with electromigration analysis in interconnects, this paper proposes an improved Graph Convolutional Network (GCN) augmented with edge feature aggregation and an attention mechanism. In deep learning, GCNs are a type of neural network that performs convolution operations on graph-structured data. They update node representations at each layer by considering the local neighborhood information of the nodes, thereby capturing key information within complex graph structures and learning features inherent to the graph. Additionally, the interconnection structure in circuits aligns with the graph structure utilized by GCNs, making GCNs well suited for the study of interconnection structures. The improved GCN model is capable of better capturing important information within the graph structure and is well suited for interconnect structures characterized by multiple edge features, which enables more accurate predictions of hydrostatic stress distribution within the interconnects. The proposed model was compared with the standard GCN model and exhibited a superior accuracy. Additionally, compared to the traditional finite element method, a significant improvement in computational speed was achieved.

## 2. Methods

In this section, the physical-based model of the EM analysis utilized in this study is first introduced. Subsequently, the GCN model and the methods employed for its enhancement are described.

### 2.1. Physical-Based Model of Electromigration

**Figure 1:**
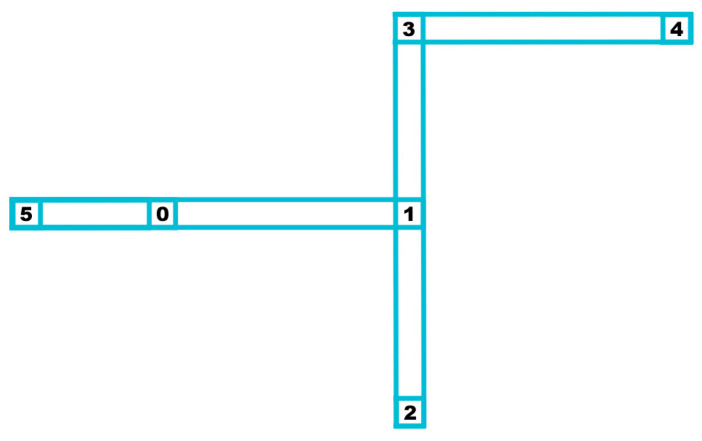
An interconnect with six nodes and five branches, numbers in the figure represent the sequence numbers of the nodes.

The electromigration (EM) effect is caused by the movement of electrons within a conducting material, transferring momentum to the metal ions of the conductor. This causes the ions to gradually migrate in the direction opposite to the electric field, leading to atomic diffusion and loss within the conductor. Under the influence of EM, hydrostatic stress develops within the metal wires. When this stress reaches a critical point, atomic migration occurs. Over time, atoms continuously flow from the cathode to the anode, resulting in the accumulation of atoms at the anode and the creation of voids at the cathode, leading to the failure of circuit interconnects. Therefore, the primary focus of EM analysis is the distribution of hydrostatic stress within the interconnects. Figure 1 is an example of an interconnect with six nodes and five branches.

To describe the process, the physics-based analytical model, which takes into account void nucleation and the kinetics of void size evolution, was originally proposed by Korhonen et al. and has since been advanced by subsequent researchers. For a generic interconnect wire consisting of *n* nodes, inclusive of *p* interior junction nodes denoted as $x_{r}\in {(x}_{r1},x_{r2},\ldots ,x_{rp})$ and *q* block terminals denoted as $x_{b}\in {(x}_{b1},x_{b2},\ldots ,x_{bp})$, the distribution of hydrostatic stress σ(x,t) along the wire is characterized by the Korhonen equation as follows [10,13,19]:

(1) ∂σijx,t∂t=∂∂xκij∂σijx,t∂x+GijBC:σij1xi,t=σij2xi,t,t>0BC:∑ijκij∂σij(x,t)∂x x=xr+Gij·nr=0,t>0BC:κij∂σij(x,t)∂x x=xb+Gij=0,t>0IC:∂σijx,0=σij,T

where *i* denotes a branch connected to nodes *i* and *j*, while $n_{r}$ signifies the unit inward normal direction of the interior junction node *r* on branch *ij*. The value of $n_{r}$ is +1 for the right direction and −1 for the left direction of the branch, assuming $x_{i}{<x}_{j}$. $G=\frac{Eq^{*}}{\Omega }$ represents the electromigration driving force, and $\kappa =\frac{D_{a}B\Omega }{k_{B}T}$ symbolizes the stress diffusivity. *E* is the electric field, and $q^{*}$ is the effective charge. $D_{a}=D_{0}exp(\frac{-E_{a}}{k_{B}T})$ is the effective atomic diffusion coefficient, with $D_{0}$ as the pre-exponential factor. *B* denotes the effective bulk elasticity modulus, Ω is the atomic lattice volume, $k_{B}$ is Boltzmann’s constant, *T* is the absolute temperature, and $E_{a}$ is the EM activation energy. Lastly, $\sigma_{T}$ is the initial thermally induced residual stress. In the equation, there are three boundary conditions (BCs) and one initial condition (IC). The first boundary condition states that the hydrostatic stress is continuous at the interface between the two segments of the interconnect, meaning that the values at this location are equal. The second boundary condition indicates that the atomic flux at the interface should satisfy continuity. The third boundary condition specifies that the atomic flux at the boundary is equal to zero. The initial condition indicates that the hydrostatic stress is zero when t=0.

A solution for the equations in simple interconnects has been proposed using different methods, but it has been shown that obtaining solutions for more general interconnects is difficult using traditional methods [26].

### 2.2. Graph Convolutional Network

A Graph Convolutional Network is a type of graph neural network model. Over the past few decades, researchers have dedicated their efforts to studying how to perform convolution operations on graphs [27]. It was not until 2016 that Kipf et al. [28] first proposed the use of GCNs for semi-supervised node classification tasks. Similar to previous Convolutional Neural Networks (CNNs), GCNs serve as feature extractors. However, the distinction lies in the ability of GCNs to extend to graph data, significantly broadening their application scope. GCNs have been employed in numerous fields such as text classification [29], image classification [30] and regression [31], with their impressive performance demonstrating their potential. The following section will introduce the foundational model of GCNs, upon which subsequent enhancements will be based.

**Figure 2:**
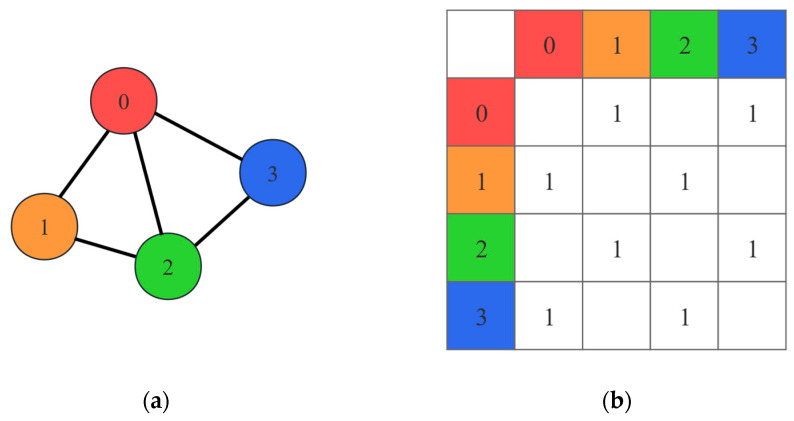
(**a**) A 4-node graph in GCN. Black lines and numbers represent edges and sequence numbers of the nodes respectively; (**b**) adjacency matrix of the 4-node graph.

The graph should consist of an adjacency matrix  A, a node feature matrix $\;X^{v}$ and an edge feature matrix $\;X^{e}$. The adjacency matrix represents the connections between nodes in the graph structure, the point features represent the values corresponding to the information of each node in the graph, and the edge features represent the values corresponding to the information of each edge in the graph. Given a graph with a node feature matrix, GCNs fundamentally aggregate information from the neighboring nodes of each node and feed it into the neural network. The propagation formula for GCNs is:

(2) $$H^{\left(l+1\right)}=\sigma ({\tilde{D}}^{-\frac{1}{2}}\tilde{A}{\tilde{D}}^{-\frac{1}{2}}H^{\left(l\right)}W^{\left(l\right)})$$

where $\tilde{A}=A+I_{N}$, with $I_{N}$ being the n-dimensional identity matrix. A is the adjacency matrix, which represents the connectivity of the nodes in the graph. Figure 2 is an example of the formation of an adjacency matrix for a graph consisting of 4 nodes.

Since the diagonal elements of the adjacency matrix A are all zero, the information of the node itself is ignored when aggregating information by multiplying with the feature matrix H. This operation allows for the feature of the node itself to be included in the calculation.

**Figure 3:**
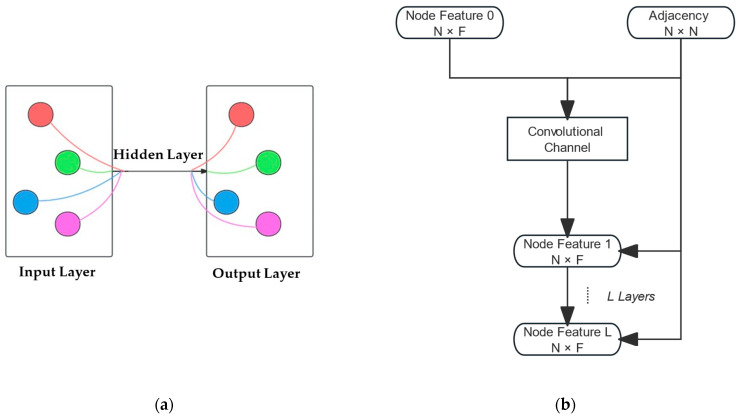
(**a**) A schematic diagram of a GCN model; (**b**) structure of layers in a classical GCN model.

$\tilde{D}\;$ is the degree matrix of $\tilde{A}$, i.e., the number of nodes each node is connected to, and its structure is a diagonal matrix. To prevent the aggregation process from being overly biased towards nodes with more edges, $\tilde{A}$ needs to be normalized, resulting in the normalized symmetric matrix ${\tilde{D}}^{-\frac{1}{2}}\tilde{A}{\tilde{D}}^{-\frac{1}{2}}$. $H^{\left(l\right)}$ represents the activation function in the l-th layer of the neural network. $W^{\left(l\right)}$ denotes the feature matrix. Figure 3 shows a graphical representation of a GCN.

As can be seen, GCNs do not alter the structure of the graph itself, and the information regarding nodes and connections remains unaffected. During the convolution process, node features are continuously updated. GCNs can be layered, and when there is l hidden layer, it can be represented as follows:

(3) $$Input\;layer:h_{v}^{\left(0\right)}=x\_vFirst\;hidden\;layer:h_{v}^{\left(1\right)}=ReLU({\tilde{D}}^{-\frac{1}{2}}\tilde{A}{\tilde{D}}^{-\frac{1}{2}}h_{v}^{\left(0\right)}W^{\left(0\right)})\;\ldots \;lth\;hidden\;layer:h_{v}^{\left(l\right)}=ReLU({\tilde{D}}^{-\frac{1}{2}}\tilde{A}{\tilde{D}}^{-\frac{1}{2}}h_{v}^{\left(l-1\right)}W^{\left(l-1\right)})$$

Here, we assume that the non-linear activation function is ReLU. After computing through l hidden layer, we can obtain the feature vector $h_{v}^{\left(l\right)}$, which contains the information we desire. Depending on the task requirements, different operations on this vector can achieve different task objectives.

In this case, it can be observed that the interconnects possess the characteristics of graph data required by GCNs: irregularity, a network containing different numbers of nodes and different nodes having different neighbors. This makes the application of GCNs for the EM analysis of interconnects a feasible approach.

### 2.3. Improving GCN with Edge Features and Attention

In order to adapt the GCN model better to the topological structure of our interconnect graphs and to enhance its performance, the methodologies delineated in this chapter are applied to refine the GCN model.

#### 2.3.1. Edge Feature Aggregation

It is obvious that edge features hold significant importance within the structure of our interconnect graphs, considering that input features such as the interconnect length, interconnect width and current density are all attributes of the edges. However, GCNs are not inherently adept at utilizing edge features within a graph [32]. Traditional GCN models usually only consider convolution operations on node features based on the adjacency matrix, ignoring the presence of edge features. If directly applied to our task, important information such as the interconnect length, interconnect width and current density will be ignored, which leads to difficulties for the model to accurately capture the features in the graph, thus failing to predict accurate results. This necessitates modifications to the GCN architecture to incorporate edge features into the analysis.

**Figure 4:**
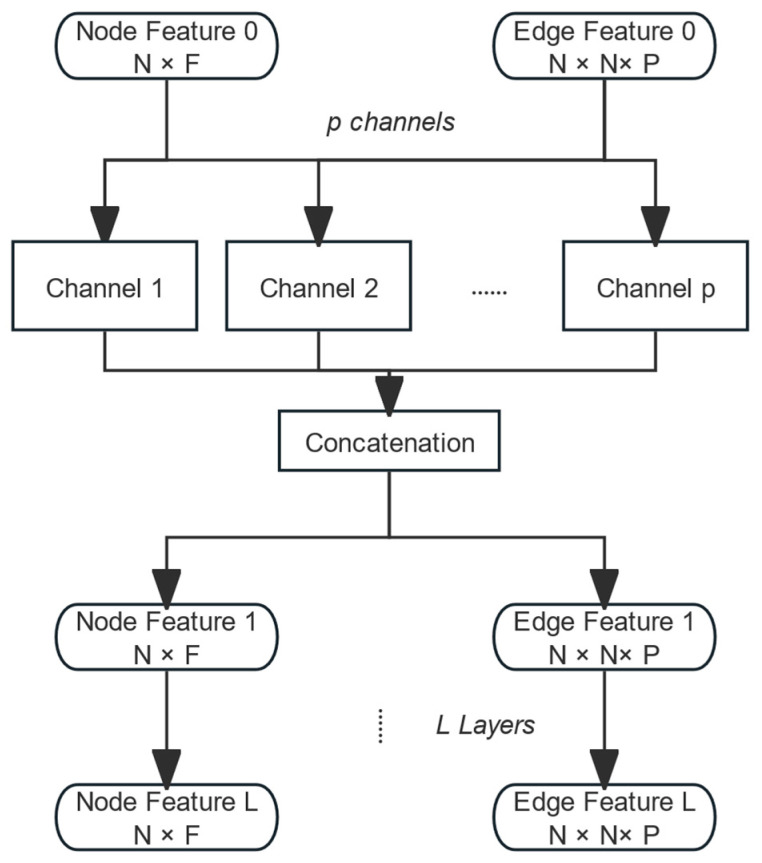
Structure of layers of the edge feature aggregation convolutional model.

To enhance the model’s capacity to utilize edge features, it is necessary to modify and augment the convolutional layers to accommodate the edge attributes. Within a GCN layer, there is typically a single node feature matrix H with which we aim to incorporate the corresponding edge features. We define the feature vector X as being aggregated from the edge feature vectors of the $i^{th}$ node’s neighboring nodes, denoted as $\;(X_{j},j\in N_{i})$, where $N_{i}\;$ is the set of all the neighbors of i. The aggregation operation is described as follows:

(4) $$X^{\left(l\right)}=\sigma \left[{∥}_{p=1}^{P}(E_{..p}X^{\left(l-1\right)}W^{\left(l\right)})\right]$$

where σ is the non-linear activation function employed. $E_{..p}$ represents the $p^{th}$ channel of the edge feature matrix, and $W^{\left(l\right)}$ is the weight matrix. In our task, multiple edge features are included, making it necessary to aggregate all edge features through a multi-channel approach. Subsequently, these aggregated features are merged via a concatenation operation. Thus, the neural network architecture is shown in Figure 4.

#### 2.3.2. Multi-Head Attention Mechanism

In GCNs, weights between different features are assumed to be the same by default. However, this approach is not suitable for our interconnect structure, as the factors influencing the hydrostatic stress do not possess the same weight. Therefore, we need to introduce an attention mechanism [33] to enhance the model’s capacity to learn from features with varying weights. The attention mechanism allows the neural network to focus on relevant parts when processing input data. By introducing the attention mechanism, the neural network can autonomously learn and selectively focus on important information in the input, thereby improving the model’s performance and generalization capabilities.

Among the various attention mechanisms, the multi-head attention mechanism can further enhance the model’s expressive power and generalization ability. It does this by using multiple independent attention heads to calculate attention weights separately, and then concatenating or weighting their results to achieve a richer representation. To incorporate the multi-head attention mechanism, we have the original definition:

(5) $$a_{ij}=\frac{\exp\left(a(a^{T}[WX_{i}∥WX_{j}])\right)}{\sum_{k\in N(i)\cup i}^{\;}\exp\left(a(a^{T}[WX_{i}∥WX_{j}])\right)}$$

(6) $$X^{\left(l\right)}=\sigma \left[{∥}_{p=1}^{P}a_{..p}^{l}W^{\left(l\right)}X^{\left(l-1\right)})\right]$$

where *a* represents the attention scores, *W* denotes the learnable parameters, and j signifies the feature vector of neighboring nodes. $X^{\left(l\right)}$ is the $l^{th}$ feature matrix. To incorporate edge features, the algorithm for calculating the attention coefficients is adjusted, adding computations for edge features, leading to the following equations:

(7) $$a_{i,j}=\frac{\exp\left(a(a^{T}[WX_{i}∥WX_{j}])\right)E_{ijp}}{\sum_{k\in N(i)\cup i}^{\;}\exp\left(a(a^{T}[WX_{i}∥WX_{j}])\right)E_{ijp}}$$

(8) $$X^{\left(l\right)}=\sigma \left[{∥}_{p=1}^{P}(a_{..p}^{l}E_{..p}X^{\left(l-1\right)}W^{\left(l\right)})\right]$$

**Figure 5:**
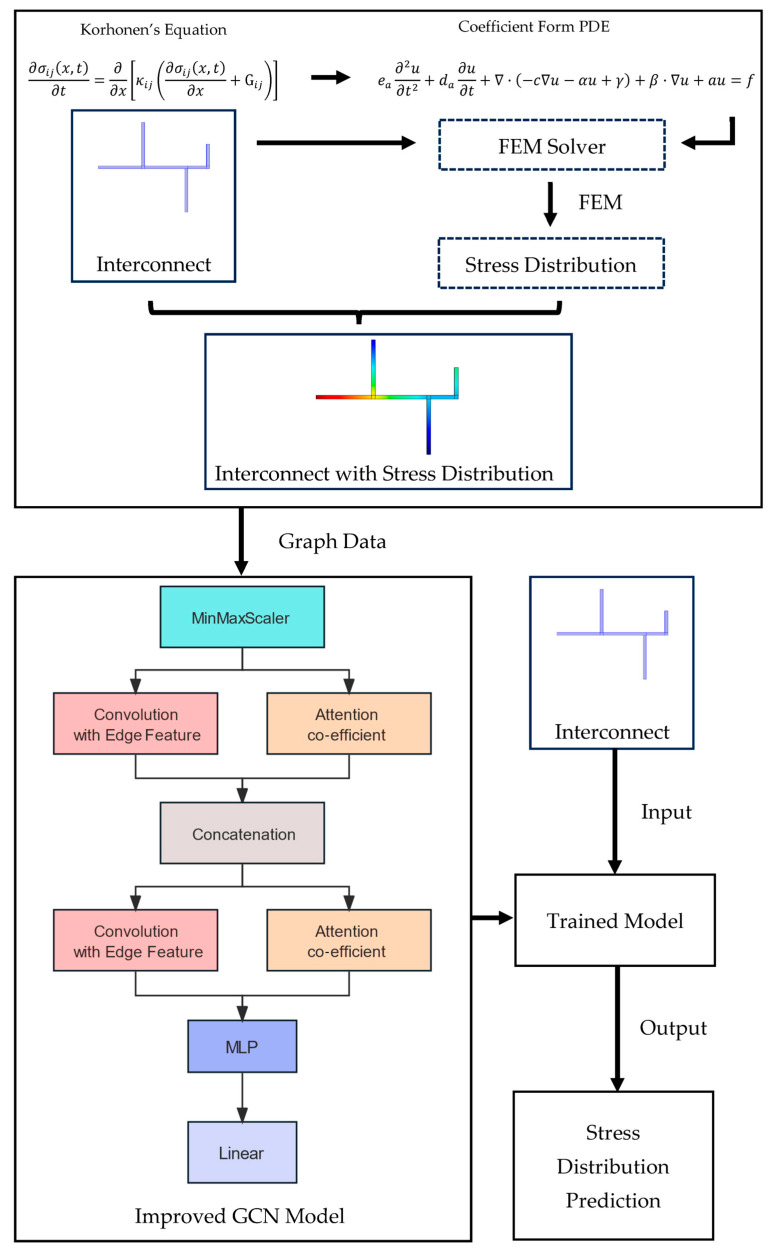
Full process of the EM analysis model.

By introducing the mechanisms mentioned above, we can obtain an enhanced GCN model suitable for EM analysis. In the process, a certain number of interconnect structures are obtained and imported into an FEM solver to determine the distribution of hydrostatic stress, where Korhonen’s equation can be seen as coefficient-form PDEs to be calculated in an FEM solver. This results in a set of interconnects that incorporate the distribution of hydrostatic stress and can be viewed as graph data with inputs and outputs. As introduced in the previous chapter concerning GCNs, such structures can be regarded as graph data within graph neural networks. The proposed improved GCN model is then utilized to train the dataset constructed from graph data. The trained model is then capable of predicting the hydrostatic stress distribution within the interconnects. The full process is depicted in Figure 5:

## 3. Experiment and Results

### 3.1. Data Preparation

#### 3.1.1. Establishment of the Dataset

Based on the introduction of the EM physical analysis model and the GCN model, it is evident that the task of building a dataset is to obtain the corresponding graph data of the interconnect model. As mentioned earlier, the interconnect model consists of metallic interconnects with multiple nodes and branches, which is the primary focus of Korhonen’s equation. The interconnection model studied in this paper primarily involves copper interconnections. We need to obtain the ground truth values from the FEM solver to serve as part of the graph data for training the model.

Initially, we can construct a model of an interconnect branch in commercial finite element physical software (COMSOL Multiphysics 5.5) and derive the stress based on the partial differential equation. The physical parameters used can be seen in Table 1, along with the input variables and output variables for the model, which are shown in Table 2.

Under the given parameters, we need to analyze the hydrostatic stress distribution of interconnects using FEM solver. As described earlier, we first set the global definitions for the model and import the interconnects into an FEM solver based on their sizes and structures. Next, we need to add equations to the model, as the FEM solver does not have built-in physical fields that can be directly applied to our task. Therefore, we utilize the general coefficient-form PDE module for the calculations. As shown in Figure 5, by comparing the coefficients from Korhonen’s equation with those in the coefficient-form PDE, we can match the coefficients in Korhonen’s equation to the corresponding parameters in the FEM solver. After comparing the coefficients, the equation input into the FEM solver is as follows:

(9) $$e_{a}\frac{\partial^{2}u}{\partial t^{2}}+d_{a}\frac{\partial u}{\partial t}+\nabla \cdot \left(-c\nabla u-\alpha u+\gamma \right)+\beta \cdot \nabla u+au=f\;\nabla =\left[\frac{\partial }{\partial x},\frac{\partial }{\partial y},\frac{\partial }{\partial z}\right]\;c=\frac{D_{a}B\Omega }{k_{B}T}\;\;\;\alpha =0\;\;\;f=0\;\;\;e_{a}=0\;\;\;d_{a}=1\;\;\;a=0\;\;\;\beta =0\;\;\;\gamma =-\frac{ceZ\rho j}{\Omega }$$

**Figure 6:**
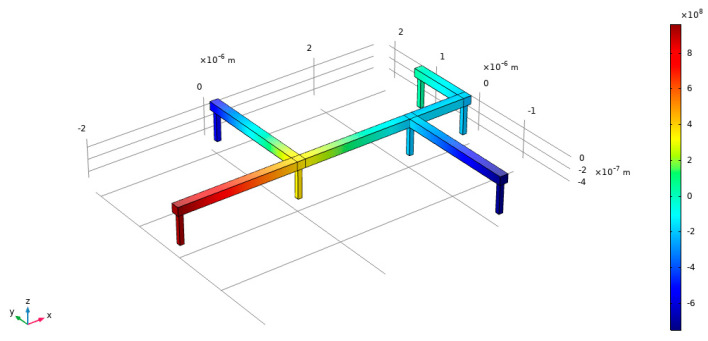
Hydrostatic stress distribution of a multi-branch interconnect from FEM solver.

Based on the current density in different branches, we can obtain various coefficients that correspond to the coefficients in their respective branch equations, allowing us to solve them using the FEM solver. Figure 6 illustrates the hydrostatic stress distribution of a multi-branch interconnect obtained using the FEM solver:

It can be observed that under the given conditions, the variables that affect the hydrostatic stress distribution of the interconnect are the current density *J*, the length of the interconnect *L*, the width of the interconnect *W* and the structure of the interconnect itself. Because in practical applications, the hydrostatic stress generated by the EM effect is mainly used to analyze and optimize the structure at the circuit vias(that is, the node in the graph structure), to simplify the problem, we set the output as the hydrostatic stress at each node. Hence, we can obtain the input and output of the model from the FEM solver.

Consequently, we can establish a directed graph G=(V,E) which is composed of a set of nodes V and a set of directed edges E. The edge feature vectors for the input incorporate the current density *J*, length *L* and width *W* for each edge in E, where *J* assumes a positive value when the current flows from node v to node u  (assuming u>v), and a negative value for the reverse direction. The node feature vector for the output corresponds to the hydrostatic stress.

To acquire a sufficient number of training sets, we devised an algorithm for generating interconnect trees. This algorithm randomly generates nodes ranging from 3 to 32 in number and creates topologies in accordance with the structural requirements of the circuit interconnects. It also assigns random lengths, widths and current densities to each branch. The generated data are then stored in four 32 × 32 matrices, namely the adjacency matrix, length matrix, width matrix and current density matrix. Subsequently, the 40,000 generated structures are imported into COMSOL to compute the hydrostatic stress, thus obtaining the ground-truth hydrostatic stress distribution.

#### 3.1.2. Preprocessing of Dataset

To facilitate model training, it is necessary to preprocess the data. The data obtained in Section 3.1.1 exhibit large magnitudes (in our dataset, the hydrostatic stress can reach up to the order of $10^{9}$), which is excessively large for our model. Therefore, we need to perform feature scaling on these data. For the dataset in this study, given that the parameters of the circuit interconnects do not exhibit extreme outliers and the overall data range is relatively stable, normalization is more suitable for data preprocessing. In this paper, we employ min–max normalization, the formula for which is as follows:

(10) $$x^{\prime }=\frac{x-min(x)}{\max\left(x\right)-min(x)}$$

Here, min(x) and max(x) represent the minimum and maximum values of the input sample data, respectively.

To train and validate our model, the samples were randomly shuffled, and 80% of the dataset was randomly selected to serve as the training set, with the remaining 20% allocated as the test set. In this paper, Pytorch was utilized as the deep learning framework, and CUDA was employed to accelerate the training process. Table 3 displays the parameters adopted during model training:

### 3.2. Evaluation of the Regression Task

In our task, the network model is employed for node feature regression. The Coefficient of Determination ($R^{2}$ score) and Root Mean Square Error (RMSE) are adopted as performance metrics. For the $R^{2}$ score, values closer to 1 indicate predictions that more closely approximate the true values. Conversely, for RMSE, lower values signify predictions that are closer to the actual values. Below are the mathematical expressions for the $R^{2}$ score and RMSE:

(11) $$R^{2}=1-\frac{\sum_{i=1}^{n}{\left(y_{i}-\hat{y_{i}}\right)}^{2}}{\sum_{i=1}^{n}{\left(y_{i}-\bar{y_{i}}\right)}^{2}}$$

(12) $$RMSE=\sqrt{\frac{1}{n}\sum_{i=1}^{n}{\left(y_{i}-\hat{y_{i}}\right)}^{2}}$$

where $y_{i}$ is the actual value of the output variable, $\hat{y_{i}}$ is the predicted value of the output variable, and $\bar{y_{i}}$ is the average value of the output variable. By analyzing the $R^{2}$ score and RMSE, we can compare the performances of different models in this prediction task.

### 3.3. Comparative Results

During the training process, the program utilizes loss.backward() to compute the gradients of the loss function with respect to the model parameters, thereby implementing the backpropagation algorithm. Subsequently, the optimizer.step() function is used to update the model parameters, allowing the model to gradually approach the optimal solution. For the regression task in this study, it is essential to set an appropriate loss function and optimizer to meet the model’s requirements and enhance its performance. We chose the Mean Square Error (MSE) as the loss function for the regression task and Adam as the optimizer.

To compare the performance of our proposed model with the existing models, we trained each model on our dataset and obtained the $R^{2}$ scores and RMSE for each, as shown in Table 4. Model A is the basic GCN model, Model B is the GCN model enhanced with the attention mechanism, Model C is the GCN model augmented with edge feature-enhanced aggregation, and Model D (the final model proposed in this paper) is a GCN model that incorporates both edge feature-enhanced aggregation and an attention mechanism. In both Model A and Model B, edge features are aggregated into node features, since GCNs do not directly process edge features. It should be noted that the RMSE values presented have been normalized and, thus, do not reflect the metrics on their original scale.

The results indicate that our proposed Model D exhibits a superior performance in terms of both the $R^{2}$ score and RMSE, demonstrating its enhanced capability in this regression task. It is evident that both edge feature-enhanced aggregation and the attention mechanism positively impact the model’s learning ability, given that compared to the basic GCN model, Model B and Model C improved the $R^{2}$ score by 1.7% and 1.4% and the RMSE by 6.3% and 8.5%, respectively. Meanwhile, Model D, which combines both improvements, increased the $R^{2}$ score by 2.7% and the RMSE by 15.0%.

**Figure 7:**
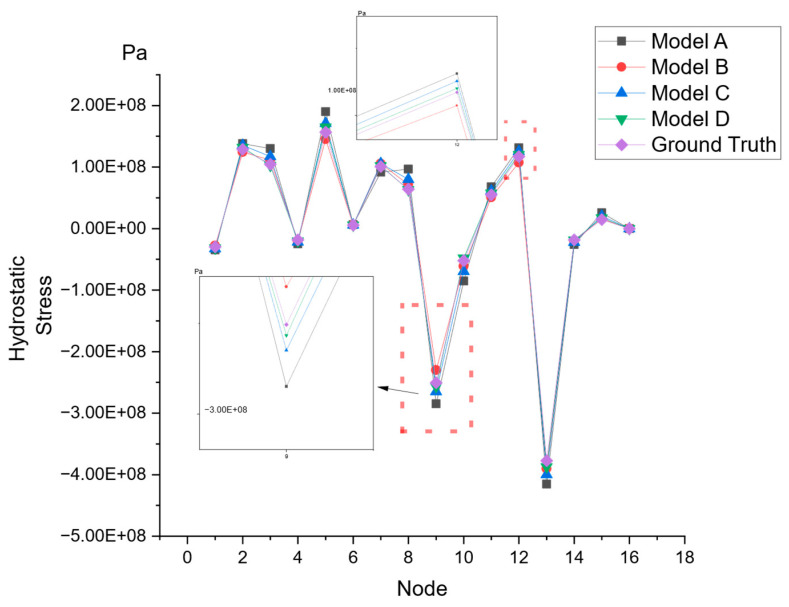
Prediction of a random interconnect from the models listed above.

Next, we randomly selected a branch with sixteen nodes and used the trained models to predict its stress distribution, as shown in Figure 7. Clearly, our proposed Model D is the closest to the ground truth in its predictions.

Lastly, the time taken for inference was evaluated. A set of 100 random interconnections can be used for testing to obtain the average inference time. The time taken is presented in Table 5

From the table, it can be seen that deep learning methods provide an order of magnitude speed advantage in inference compared to COMSOL. Among deep learning models, the basic GCN model exhibits the fastest inference speed due to its simple structure. After incorporating additional mechanisms, there is a slight increase in inference time. Specifically, our final model, D, only showed an approximately 3.1% increase over the baseline model. Considering the substantial increase in inference accuracy, our proposed model’s performance is in a reasonable range.

## 4. Conclusions

In this work, in order to solve the hydrostatic stress in circuit interconnects, we analyzed the physical model of EM hydrostatic stress and constructed a dataset for EM analysis. Edge feature aggregation and attention mechanisms are integrated into the basic GCN model for our task. To adapt the model for regression tasks, MLPs and linear layers are introduced to learn complex features of the data and produce outputs for gradient descent optimization. The improved GCN model can better take advantage of edge features and capture the importance of features more effectively. This allows the model to better predict features of graph topologies such as circuit interconnects, enabling the rapid and accurate determination of stress values in the given interconnect nodes. This greatly improves the possibility of full-chip EM analysis to acquire the hydrostatic stress distribution. The method is beneficial to optimize routing in the backend of integrated circuits, reducing the impact of electromigration effects and enhancing reliability.

## Figures and Tables

**Table 1:** Physical parameters.

Feature	Definition	Value	Unit
Ω	Lattice constant	$8.78\;\times \;10^{-30}$	$m^{3}$
$D_{0}$	Pre-diffusion index factor	$7.56\;\times \;10^{-5}$	$m^{2}/s$
h	Heat transfer coefficient	5	$W/{(m}^{2}\times K)$
T	Temperature	293.15	K
Q	Heat consumption rate	$10^{5}$	$W/m^{2}$
B	Bulk elastic modulus	$10^{11}$	Pa
Z	Effective valence charge	10	1
ρ	Cu resistivity	$1.67\;\times \;10^{-8}$	Ω×m
e	Electron charge	$1.6\;\times \;10^{-19}$	C
t	Research time	$10^{5}$	t
$k_{B}$	Boltzmann constant	$1.38\;\times \;10^{-23}$	J/K
$E_{a}$	Diffusion activation energy	$1.2817\;\times \;10^{-19}$	J

**Table 2:** Input variables and output variables in the model.

Sort	Feature	Definition	Type	Unit
Input variables	A	Adjacency relation	Edge	/
	L	Length	Edge	$A/m^{2}$
	W	Width	Edge	μm
	J	Current density	Edge	μm
Output variables	σ	Hydrostatic stress	Node	Pa

**Table 3:** Parameters for model training.

Parameters	Value
Input resolution	32 × 32 × 4
Epoch	1000
Learning rate	0.0001
Batch size	64

**Table 4:** Performance evaluation table for the models.

Model	*R*^2^ Score	RMSE
A	0.94719	0.07578
B	0.96291	0.07098
C	0.96089	0.06931
D	0.97236	0.06445

**Table 5:** Time taken for inference for different methods.

Methods	Time (s)
Model A	0.05834
Model B	0.05912
Model C	0.05941
Model D	0.06015
FEM solver	~9

## Data Availability

Data are contained within the article.
